# Supplementary material for: From sequence to enzyme mechanism using multi-label machine learning
Source: BMC Bioinformatics. 2014 May 19;15:150. doi: 10.1186/1471-2105-15-150 (PMC4229970; doi:10.1186/1471-2105-15-150)
Supplement: Additional file 2 — Java code of ml2db. Additional file ml2db_code.tar.gz contains the Java source code to run the multi-label machine learning experiments and save the results to database. The code’s Javadoc is included. [file 1471-2105-15-150-S2.zip › additional file 2/ml2db/ml2db/doc/main/ml2db/mulan/learn/traintest/package-tree.html]

main.ml2db.mulan.learn.traintest Class Hierarchy


JavaScript is disabled on your browser.


- Overview
- Package
- Class
- Use
- Tree
- Deprecated
- Index
- Help

- Prev
- Next

- Frames
- No Frames

- All Classes

# Hierarchy For Package main.ml2db.mulan.learn.traintest

Package Hierarchies:

- All Packages

## Class Hierarchy

- java.lang.Object
  - main.ml2db.mulan.learn.crosseval.CrossExperimenter
    - main.ml2db.mulan.learn.traintest.TrainTestExperimenter
      - main.ml2db.mulan.learn.traintest.TrainTestExperimenterSerialized
  - main.ml2db.mulan.learn.crosseval.CrossInputManager
    - main.ml2db.mulan.learn.traintest.TrainTestInputManager
  - mulan.evaluation.Evaluator
    - main.ml2db.mulan.learn.evaluation.EvaluatorNoAUC
      - main.ml2db.mulan.learn.crosseval.CrossEvaluator
        - main.ml2db.mulan.learn.traintest.TrainTestEvaluator
          - main.ml2db.mulan.learn.traintest.TrainTestEvaluatorSerialised
  - uk.ac.standrews.utils.main.Initialised (implements uk.ac.standrews.utils.main.Initialisable)
    - main.ml2db.mulan.learn.crosseval.CrossOutputManager
      - main.ml2db.mulan.learn.traintest.TrainTestOutputManager
  - main.ml2db.mulan.learn.traintest.MulanTrainExample
  - main.ml2db.mulan.learn.traintest.TrainTestFullRun

- Overview
- Package
- Class
- Use
- Tree
- Deprecated
- Index
- Help

- Prev
- Next

- Frames
- No Frames

- All Classes
